# Emerging technologies in autoantibody testing for rheumatic diseases

**DOI:** 10.1186/s13075-017-1380-3

**Published:** 2017-07-24

**Authors:** Nancy J. Olsen, May Y. Choi, Marvin J. Fritzler

**Affiliations:** 1 Penn State M.S. Hershey Medical Center, 500 University Drive, Hershey, PA 17033 USA; 2 Cumming School of Medicine, University of Calgary, Calgary, AB T2N4N1 Canada

**Keywords:** Systemic autoimmune rheumatic diseases, Systemic lupus erythematosus, Antinuclear antibodies, Autoantibodies, Biomarkers, Point of care testing, Immunoassays

## Abstract

Testing for the presence of antinuclear antibodies (ANAs) is a key step in the diagnosis of systemic lupus erythematosus (SLE) and other systemic autoimmune rheumatic diseases (SARD). The standard slide-based indirect immunofluorescence (IIF) test is widely used, but is limited by a relative lack of specificity for SLE and not all SARD-ANAs are detected. Alternative immunoassays that might offer enhanced diagnostic and prognostic information have evolved, and some of these have entered clinical practice. This review summarizes the current state of ANA testing and multiplex techniques for detecting other autoantibodies, the possibility of point-of-care testing, and approaches for applications in early disease stages.

## Background

Systemic autoimmune rheumatic diseases (SARD) afflict 3–5% of the population [1] and are associated with enormous burdens on individuals and society due to poor quality of life, lower productivity, and growing direct and indirect healthcare costs [2, 3]. A hallmark of SARD is the presence of autoantibodies directed against a spectrum of intracellular autoantigens, in general referred to as antinuclear antibodies (ANAs) [4]. Often, ANAs are the only disease-specific serological markers for an underlying SARD and, as such, many have become part of classification criteria developed to provide a common basis for disease prediction and prognosis, an accurate diagnosis, disease monitoring, entry into therapeutic trials, and understanding the pathogenesis of the SARD [4–7]. While the ANA test is generally requested as a means of confirming a diagnosis and intent to treat a specific SARD, in a general practice setting it is also used as an approach to case finding [8]. Although clinicians formulate a differential diagnosis based on history and physical examination, practice analysis shows that most readily act only after receiving confirmatory or exclusionary laboratory test results [9, 10]. Hence, it is important to have an understanding of the proper use of autoantibody testing and its value, as well as limitations [4].

One of the major challenges in adopting an evidence-based approach to ANA testing is the discrepancies observed in studies that characterize autoantibodies as biomarkers of disease onset and disease activity. These discrepancies are due to a wide range of variables that include technical aspects of clearly defined methodology and assay performance, but are also due to a lack of prospective or longitudinal studies, patient selection bias, use of inconsistent definitions for disease activity, variations in nomenclature of autoantibodies, frequency of testing, and effects of therapy, which contribute to conflicting results [11, 12]. It should also be noted that disease activity, disease severity, and the ensuing irreversible tissue and organ damage should be conceptually distinguished, and measurement tools for these parameters may be different [13]. Despite these potential problems in interpreting ANA results, the clinician’s goal of making an early and accurate diagnosis as well as judging disease activity has made the practice of ordering autoantibodies widespread and frequent [14, 15]. For example, more than 90% of US rheumatologists use serial anti-dsDNA autoantibody titers to monitor disease activity in systemic lupus erythematosus (SLE) [10].

In this review, we outline the use of common and emerging technologies that are used to detect antinuclear and related autoantibodies (Table 1).

**Table 1:** Advantages of various ANA immunoassay techniques

Assay Type	Low cost	Rapid	Automated	Sensitivity	Specificity	Quantitative	Availability	Reproducibility	High throughput	Small sample volume
IIF	+	+	(++)*	++	+	++	++	+	+	–
ELISA	++	++	++	+++	++	++	++	++	++	+
LIA	–	++	++	++	++	++	++	+	+	++
ALBIA, CIA, other bead-based arrays	++	+++	+++	+++	++	+++	++	+	+++	+++
Planar array	++	++	++	++	++	+	–	–	++	++
Point-of-Care	+	+++	–	+	++	+	–	++	–	+

*Automated antinuclear antibody (ANA)-indirect immunofluorescence (IIF), antineutrophil cytoplasmic antibody (ANCA), and *Crithidia luciliae* fluorescent test (anti-dsDNA; CLIFT) used in some laboratories

*ALBIA* addressable laser bead immunoassays, *CIA* chemiluminescence immunoassay, *ELISA* enzyme-linked immunosorbent assay, *LIA* line immunoassay

## Autoantibody detection

### Indirect immunofluorescence (IIF): including automated, digital IIF

Indirect immunofluorescence (IIF) for the detection of ANAs and other autoantibodies has been in use for more than 50 years and the substrates have evolved from rodent organ sections to tissue culture (i.e., HEp-2) cells [4]. The IIF method has the advantages of providing a semiquantitative result, unlike the LE cell preparation that it displaced, and offers insights about other potential antigen reactivities by displaying staining patterns that provide a clue to the specific autoantibody targets [16, 17]. Although diagnostic technologies have evolved from low-resolution microscopy to highly automated, robotic, and digital microscopy [4, 18], IIF remains one of the most widely used screening tests for SARD [4, 19, 20]. In 2010, due to concerns about other technologies such as enzyme linked immunosorbent assays (ELISAs) and other diagnostic kits that were being increasingly used as the ANA screening test by high-throughput laboratories, the American College of Rheumatology engaged a study group which eventually published a position paper indicating that IIF on HEp-2 cells should remain the “gold standard” for the detection of ANAs [21]. While this might have been expected to encourage diagnostic laboratories to return to the IIF ANA test, there is little evidence that this has happened. Indeed, when an ANA IIF on HEp-2 substrates is used as a screening test for the wide spectrum of SARD, it is troubled by both false positive and false negative results and other limitations that may mislead the clinician [4, 9, 22]. Despite decades of effort, standardization of the IIF ANAs has been a challenge due to intermanufacturer variations in the production of the HEp-2 substrate, characteristics of the secondary antibody, and expertise and subjectivity of the person(s) performing and reading the slides [20, 23]. In addition, the lack of consensus on ANA pattern nomenclature was a major issue which is now being resolved due to the efforts of the International Consensus on ANA Patterns (ICAP) group [24].

Novel approaches to improving the sensitivity and specificity of the IIF ANA test include both ‘knock-in’ and ‘knock-out’ gene technology. In the former, to improve the ability to detect anti-SSA/Ro60 on HEp-2 substrates, Tom Gordon at Flinders University in Adelaide developed a ‘knock-in’ cell line where the corresponding Ro60 cDNA was transfected and overexpressed in HEp-2 cells [25], an approach that led to the HEp2000 substrate (ImmunoConcepts, Sacramento, CA, USA) that is used in some diagnostic laboratories [26]. More recently, a ‘knock-out’ substrate has been developed as an approach to detecting antibodies to dense fine speckles (DFS70) antigen (discussed later) during the screening ANA IIF test. In this substrate, ‘normal’ HEp-2 cells are admixed with HEp-2 cells that have the DFS70 gene knocked out at a ratio of 9:1, allowing detection of DFS70 [27]. It must be emphasized that this approach only detects sera with monospecific anti-DFS70 autoantibodies and still requires wider interlaboratory validation.

### ELISA

ELISAs used for the detection of autoantibodies in SARD sera came into wide use in the 1980s and have become a mainstay in many diagnostic laboratories. In this setting, they have two main uses: 1) as a quantitative screening test to detect a wide variety of ANAs without indicating the specificity of a positive test result; and 2) as an approach to identifying specific autoantibody targets. Standard available ELISA panels used for SARD diagnosis usually include SSA/Ro, SSB/La, Sm, Sm/RNP, Scl-70/topoisomerase I, Jo-1, anti-centromere, and anti-cardiolipin, and others are increasingly available. ELISA results are reported as quantitative units rather than as serum dilutions as for IIF.

During the last decade, different strategies have been utilized to develop, evaluate, and commercialize several ANA screening ELISAs in an attempt to eliminate the more labor-intensive and subjective ANA IIF assay (reviewed in [28, 29]). The majority of these ANA screening ELISAs make use of blends of purified proteins derived from native sources (i.e., cells or tissues) and/or synthetic, recombinant technologies [30]. The composition of these preparations is as diverse as the various manufacturers producing them but, in general, they contain many of the key target autoantigens associated with SARD. Despite remarkable progress, they are attended by technical challenges of combining different autoantigens in a single assay, and reactivity to some SARD-related autoantibody targets can be missed even if the autoantigens are contained in the mixtures [31]. This is probably due to differences in physicochemical properties of individual autoantigens and it is suspected that some antigens also bind to other targets in the same mixture, resulting in a masking effect that may lead to poor autoantibody binding. Although ELISAs that are widely used to detect individual autoantibodies generally perform much better than ELISAs that use antigen mixtures, some limitations of autoantibody binding to exposed epitopes on the autoantigens that are absorbed to the plastic microtiter plates are observed in those assays as well.

### Line immunoassays

Line immunoassays (LIAs) are considered a variation of immunoblots and dot-blots [4]. A wide assortment of LIAs are commercially available and they are typically used to identify not just ANAs, but also other autoantibodies [4, 32, 33]. In some settings, LIAs also have been used to screen for disease-specific autoantibodies that are seen in SARD, paraneoplastic, and autoimmune liver diseases, and one standard kit includes 15 different SARD-related autoantibody specificities [4, 33]. One limitation of LIAs is that they are not as adaptable to high-volume and high-throughput laboratories, although components of the technology have been automated making them more suitable in these settings. The reactivity of autoantibodies to specific LIA targets can be quantitated by densitometry. Despite their ease of use, LIAs have other drawbacks, including the lack of sensitivity and specificity for certain autoantibodies [32, 33].

### Addressable laser bead immunoassays

Addressable laser bead immunoassays (ALBIAs) were introduced approximately 15 years ago as a rapid, economical, quantitative, and reliable technology to detect autoantibodies directed to a range of target autoantigens in SARD sera (reviewed in [34]). Multiplexed bead-based technologies have been adopted by several diagnostic kit manufacturers based on two main technology platforms, Luminex and BioFlash chemiluminescence immunoassay (CIA) [34–38]. Similar to ELISA and LIA kits, ALBIA/CIAs are used for the detection of autoantibodies to a variety of autoantigens, [39–43] and the number and characteristics of autoantigens employed in ALBIA/CIAs and their performance vary between kit manufacturers. Hence, it is important to be aware of the performance characteristics of the specific ALBIA/CIA kits used by diagnostic laboratories. A study comparing ALBIA, ANA IIF and different ELISA assays for the detection of ANA found that 7.4% of healthy donors had a positive test and, of those, the majority had a speckled IIF staining pattern [41], raising the question whether these ‘false positive’ ANAs detected by IIF might represent autoantibodies to DFS70, a marker that has been shown to be more common in healthy or nonSARD patients than in SARD patients [44, 45]. A recent study of a large international inception SLE cohort confirmed that isolated anti-DFS70 antibodies using CIAs are rare in SLE (1.1%) [46]. Therefore, this antibody may be helpful in distinguishing ANA-positive individuals and SLE. Of note, the DFS70 nomenclature, also termed lens epithelium-derived growth factor (LEDGF), refers to the IIF staining pattern characterized by uniformly distributed fine speckles throughout the interphase nucleus and on metaphase chromatin along with the autoantigen target having a molecular mass of 70 kDa in immunoblots [47]. Although anti-DFS antibodies were first reported in interstitial cystitis and were later found to be associated with a number of other conditions, including atopic dermatitis, the current consensus is that they rarely occur in isolation in SLE, systemic sclerosis, Sjögren’s syndrome, and mixed connective tissue diseases [44].

### Multiplexed autoantibody arrays

#### Planar arrays

SLE is characterized by humoral autoimmunity and over 180 distinct autoantibodies have been associated with the disease [48]. This means that usual laboratory panels of ANA-related autoantibodies, designed to detect 10 to 15 different types, provide a view of less than 10% of the autoreactivity that may be present in a patient. SLE is therefore an ideal disease for diagnostic approaches using planar arrays that detect hundreds or more different autoantibodies.

Most of these techniques involve robotic spotting of purified autoantigen proteins or selected oligonucleotides on glass slides, although other support matrices have also been used. Investigators at Stanford University first reported, about 15 years ago, studies using an array consisting of 196 molecules corresponding to major autoantigens associated with human diseases [49]. Autoantibodies in human serum that recognize these targets are quantitatively detected by a secondary fluorescent antibody, and results show greater sensitivity than those obtained using standard ELISAs. In a more recent iteration, these investigators have developed a protein microarray with other components that are capable of detecting antibodies to soluble mediators including cytokines [50].

**Fig. 1:**
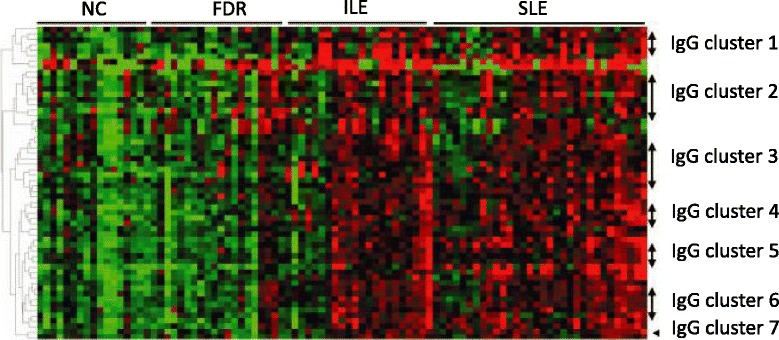
Heat map for 58 IgG autoantibody specificities analyzed on a planar array. Serum samples were obtained from normal controls (*NC*), first-degree relatives of patients (*FDR*), and patients with incomplete lupus (*ILE*), or systemic lupus (*SLE*). The normalized fluorescent intensity values were used to generate the heat map. Clustering algorithms were used to detect autoantibodies that grouped together for the tested samples. *Red* color indicates reactivities that are above the mean of all samples, *green* corresponds to values below that mean and values close to the mean are *black*. From Li et al., Clin Exp Immunol 2007 [52] with permission

A similar type of array was developed by Zhu, Li and colleagues at the University of Texas Southwestern, using known autoantigens for various human diseases [51]. Detection of autoantibodies in different classes was shown to be correlated with some types of SLE-related conditions. For example, patients with incomplete forms of lupus, designated as incomplete lupus erythematosus (ILE), were comparable to SLE patients in terms of the total quantity of serum autoreactivity, but relatively more of these autoantibodies were in the IgM class [52]. These array data were analyzed using unbiased clustering programs similar to those employed for gene expression microarrays (Fig. 1). The computer algorithms produced clusters of related autoantibodies, such as ones that reacted with structural or nuclear antigens, providing insights into how autoantibodies might be related to each other. Further insights were developed when the autoantibody array data were correlated with gene signatures expressed in the same ILE or SLE patients. Expression of the Type I interferon (IFN) gene signature that is characteristic of SLE and other autoinflammatory conditions was found to be correlated with high levels of the IgG autoantibodies. This suggests a role for IFN in driving the class switch from IgM to IgG, thus probably contributing to more active disease manifestations in the SLE patients [53].

Related findings were reported by others using a different array that detected 930 autoreactivities, split between IgMs and IgGs [54]. Patients with SLE in this study had upregulation of IgG autoantibodies, while some IgM autoantibodies were distinctly downregulated. This study also showed that SLE patients in remission had autoantibody patterns that were distinct from those of healthy controls. Serendipitously, one healthy control in this study, who initially classified within the SLE domain, subsequently developed an illness that was consistent with SLE, suggesting that the array could detect preclinical stages of the disease.

Insights into genetic effects on the autoantibody profile were provided by studies of SLE twins using arrays detecting 65 autoantigens [55]. The arrays in this analysis provided a panoramic view of IgG and IgM patterns, permitting detection of what were essentially shared IgG fingerprints or signatures in monozygotic SLE twins. While previous studies had suggested this type of association in twins, the scope of autoantibodies detected with the arrays provided the opportunity to establish a much more detailed concordance.

An advantage of the planar array approach is that only very small volumes of serum sample are required to detect hundreds of specificities (Table 1). When a fluid is not available in abundance, and especially for studies designed to explore novel specificities, this feature assumes greater importance. An example is the use of array profiling to study biomarkers in cerebrospinal fluid (CSF) [56]. Using a human proteome microarray with approximately 17,000 proteins, patients with neuropsychiatric (NP)SLE were found to have significantly more autoreactive antibodies in CSF than patients who did not have NPSLE. A network analysis of these data using the Ingenuity Pathway Analysis tool (Ingenuity System Inc, USA) showed that most of these samples were enriched in pathways related to neurologic disease; not a surprising result, perhaps, but reassuring that the method does detect physiologically relevant specificities. Furthermore, this is another demonstration of how array autoantibodies might be analyzed to give a more complete picture of overall relatedness.

In patients seen in a pediatric setting, detection of large numbers of analytes from small volume samples is also especially useful. An array displaying 140 autoantigens was used to compare pediatric patients with lupus nephritis (LN) to aged-matched healthy controls [57]. Candidate autoantigens for pediatric LN identified by cluster analyses were validated with ELISA. Specificities that were associated with LN included some that are generally known, such as C1q and dsDNA, but others that are relatively novel, such as collagens IV and X. Previous studies in adult SLE using a different autoantigen array had also shown clustering of collagen IV with dsDNA, suggesting that these independent results have broad validity [52]. Furthermore, the pediatric patients were found to have elevated levels of antibodies to B cell activating factor (BAFF), as had been described previously by this group in adult SLE [50]. The pediatric studies also attempted to develop a predictive signature for LN from a longitudinal subset of the patients, and these data suggest that persistently low nephritis scores derived from array analyses might be associated with a stable course.

Urine is a very relevant biosample to study in SLE, given the high prevalence of renal involvement and the need for sensitive and noninvasive biomarkers of LN. Array analyses of urine samples offer the potential for insights into mediators that may be specific for renal injury. One recent report utilized a commercially available investigational array of 274 human cytokines that is described as being suitable for all liquid sample types [58]. The study included serum and urine samples from SLE patients and the array findings were validated by ELISA. This approach identified adipokines, including adiponectin, resistin, and leptin, as potentially important mediators in LN and utilized pathways analysis to show relationships between different specificities. This illustrates the utility of an array approach for biomarker discovery.

The use of selected autoantigen arrays carries with it the limitation of bias for known specificities. Alternatives to the use of known autoantigens include synthetic ligands such as peptoids which have been used to create highly diverse unbiased libraries that have potential for uncovering novel specificities and biomarkers [59, 60]. Isolation of IgGs binding to arrayed peptoids in patients with SLE identified autoantibodies to RNA-binding proteins [61]. An unexpected result in this study was that one ligand bound several different autoantibodies, possibly indicating polyreactivity of these autoantibodies. These data generated an estimated specificity for an SLE diagnosis of greater than 97%, with a positive likelihood ratio of 7.45 to 28.

**Fig. 2:**
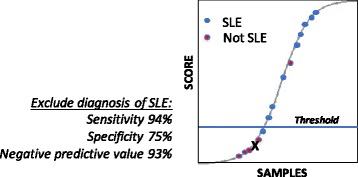
Example of array data, the SLE-key® rule-out test, that might be used to predict likelihood of an accurate systemic lupus erythematosus (SLE) diagnosis. Samples of known classification, SLE (*blue*) or non-SLE (*red*) are shown. An unknown sample in such a system would be tested to generate a score that in turn would be plotted on this curve (shown as “*X*”). Samples lower than a validated threshold (*blue line*) would have a low likelihood of an SLE diagnosis. Such data could be useful for triaging ANA-positive patients referred for rheumatology consultation (see references [62, 63])

One array platform that has been developed for clinical applications has the specific objective of excluding of the likelihood of a diagnosis of SLE [62, 63]. The low specificity of ANA testing for SLE makes this a very significant problem for clinical rheumatologists, who are confronted with screening large numbers of referred individuals, only a few of whom actually have disease [8]. A more predictive test would be welcome as a tool to prioritize patients for evaluation. For the SLE exclusion test, an array of 200 specificities, including known autoantigens and a set of proprietary oligonucleotides, was used to generate a dataset from SLE patients and healthy controls that was then analyzed by linear discriminant analysis to optimize sensitivity and specificity. A schematic of the data might be presented to the clinician, showing where the individual tested fits into the profile (Fig. 2). The cutoff chosen for a negative result, meaning SLE is unlikely, performs with a sensitivity of 94%, specificity of 75%, and negative predictive value of 93%. While the separation is imperfect, the test has potential to help overbooked rheumatologists to triage consults for possible lupus evaluations.

The strengths and weaknesses of planar autoantigen arrays have been well described (Table 2) [51, 64]. In general, the unbiased approach to collecting data on a large number of autoantibodies is a major advantage for exploratory studies. Given the relatively small number of autoantibodies detected in clinical diagnostic laboratories compared to the many known antibodies in SLE and other SARD, it is highly likely that new specificities can be found that offer greater utility for successful disease classification and for predicting flares. A major challenge is the jurisdictional (i.e., Food and Drug Administration) approval and certification of array assays for routine use in diagnostic laboratories. Clinical diagnostic applications will require reproducibility between different platforms, decreased inter- and intratest variability, standardized quantitation, and interlaboratory validation; the lupus rule-out test discussed above may have overcome some of these obstacles [51, 62].

**Table 2:** Strengths and Weaknesses of Autoantigen Array Methods

Strengths	Permits screening of many autoantigen targets simultaneously Uses very small sample volumes Can make use of stored samples May detect multiple antibody classes or subclasses Has high throughput capabilities Permits exploratory approaches to find new targets Applies unbiased analytics Carries generally lower cost per specificity than ELISA Develops insights into autoantibody clusters and relatedness
Weaknesses	Potentially difficult to optimize all targets in one array Has batch-to-batch variability Generally lacks standardization between laboratories Normalization standards are variable Has diminished sensitivity for low-affinity autoantibodies May miss autoantibodies present at low concentrations Some autoantigens are not suitable as targets Results may be semiquantitative

Detection of rare or low-affinity autoantibodies is another challenge. Addressing these issues likely will require advances in materials science in terms of developing better platforms and detection methods. An example is the use of plasmonic gold substrates with near-infrared fluorescence enhanced imaging, which has been shown to deliver a broader dynamic range and improved performance in detection of low-affinity antibodies compared to results obtained with glass slides [65]. Labeling modifications might include use of two-color antigen-binding antibody fragments (Fabs), which has been shown to enhance reproducibility of autoantibody detection [66].

#### Bead-based arrays

An alternative to the two dimensional or planar array platform is the use of beads to carry the targets, such as those in ALBIAs and CIAs, discussed above. This approach also has been used to produce an 86-specifity assay for selected autoantigens [67]. The target proteins were from commercial sources or were produced in an *E. coli* expression system. Antibody binding from diluted serum samples was measured using a fluorochrome-labeled secondary antibody, and an instrument was used to quantitate fluorescence intensity. Analysis of single autoantibodies demonstrated the expected wide range of prevalence in SLE patients (*n* = 69). These included well-known associations such as with dsDNA, but also revealed autoantibodies that were not known previously to be associated with SLE. The latter included some related to apoptosis and neutrophil extracellular traps (NETs) which have been shown to contain SLE-related autoantigens [68]. Multivariate analyses showed clear separation of healthy controls from the SLE patients, and clusters of related autoantibodies in the SLE patients were demonstrated using a heat map. A limitation of this study was that other autoimmune diseases or conditions were not included, so performance for distinguishing conditions related to SLE or subsets of SLE such as ILE or LN is unknown. The authors raised the question of whether bead arrays might have less batch-to-batch variability which would make this approach possibly preferable to planar designs for applications in clinical assays, but direct comparisons of bead and planar arrays have not been reported, so this remains speculative. Bead-based arrays do likely have some distinct advantages including reproducibility, rapid and high-throughput design, and reasonable cost.

## Detection of early disease

In the past decade, it has become clear that autoantibodies are present in the preclinical stages of SARD including SLE [69, 70]. This information has raised interest in pushing the diagnostic window earlier in the time course, prior to the development of significant morbidity or organ damage. Patients with ILE have fewer autoantibodies than SLE patients as well as lower ANA titers [71]. Studies using two-dimensional autoantigen arrays have shown that ILE patients have patterns of autoreactivity that lie intermediate between healthy individuals and those with SLE [52]. Furthermore, ratios of autoantibodies expressed in IgG and IgM classes differ, with the ILE patients showing relatively greater IgM-class autoreactivity. ILE patients are not necessarily individuals who are in early stages of lupus, but ILE is clearly a category that includes patients at high risk of progression to classifiable SLE. In one small study, 3 of 22 patients with ILE who progressed to SLE over a mean time period of 2.4 years showed significant increases in some IgG specificities detected on an array [72]. One of these was SSB, consistent with other findings indicating that this specificity increases late in the preclinical period [73]. Whether something like the SLE exclusion test array that has already been discussed [62] might help to discriminate ILE patients with and without risk of progression is an interesting possibility that has not been investigated.

## Point of care (POC) diagnostic technologies

**Fig. 3:**
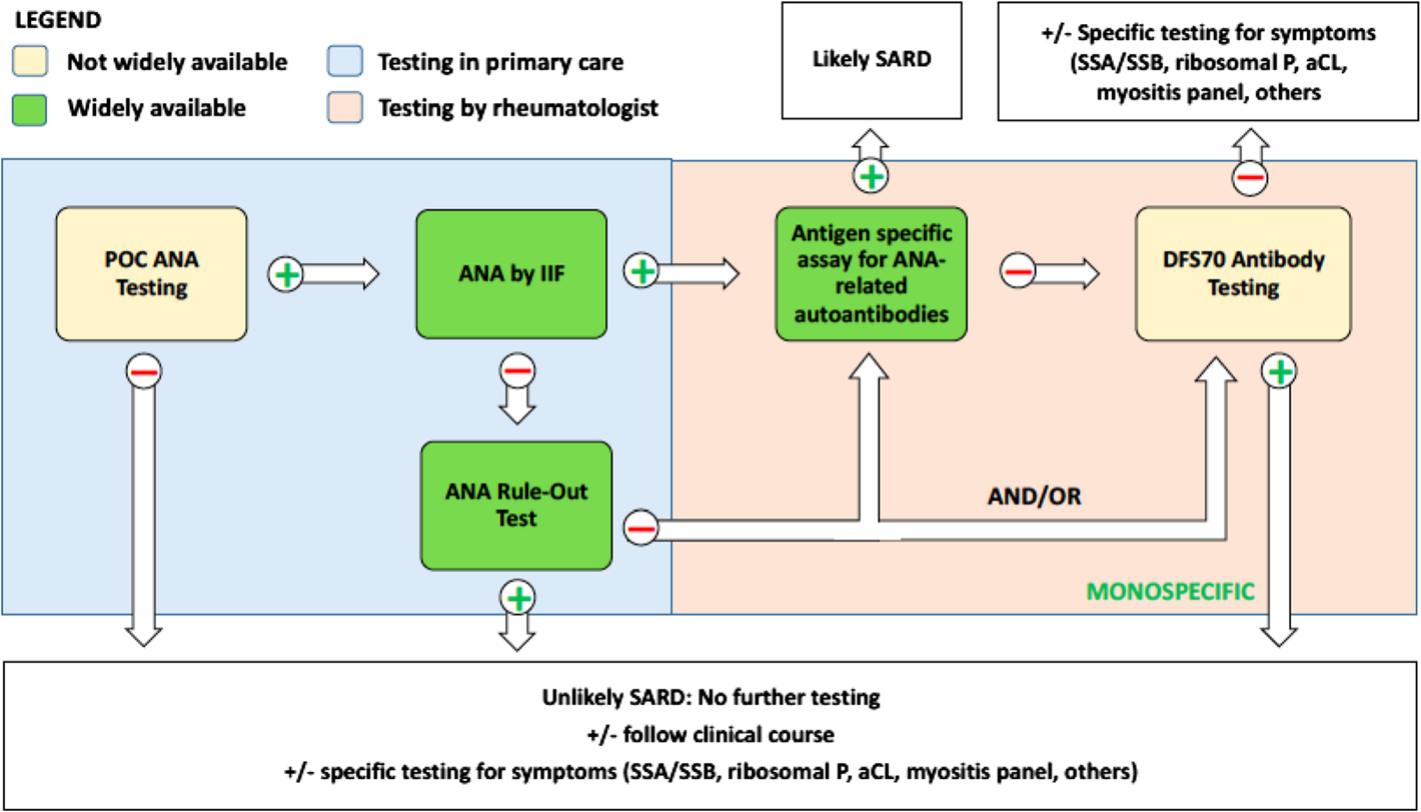
A schematic of one possible algorithm for evaluation of an SLE diagnosis using existing and proposed technologies. A point of care (*POC*) test for antinuclear antibodies (*ANA*) might be used as an office screening tool in primary care. A positive result would prompt further screening by measuring ANA by indirect immunofluorescence (*IIF*); the SLE-key® rule-out test might be also used. Positivity for ANA and a “not-ruled out” result for the SLE-key® test would then suggest a need for further autoantibody profiling using an antigen-specific assay. A negative test result for ANA, even with a “ruled-out” result on the SLE-key® test, may require further evaluation depending on the clinical scenario. DFS70 testing might be used to further improve diagnostic accuracy. The *blue box* indicates testing that might be done in primary care, with other tests being done after rheumatology referral. *Yellow boxes* indicate tests in development or not widely available; *green boxe*s indicate tests that are clinically available, at least in the USA. *aCL* anti-cardiolipin antibodies, *DFS70* dense fine speckles 70, *SARD* systemic autoimmune rheumatic diseases

The consultative and diagnostic services in rheumatology are not typically considered clinical emergencies, which require same-day diagnostic or clinical decisions. While this may hold true for certain chronic and noninflammatory conditions, it is important to appreciate that SLE, vasculopathies, and antiphospholipid syndrome can present with or develop intercurrent acute and life-threatening features which need to be diagnosed and acted upon quickly to prevent irreversible immune-mediated damage and mortality. It is in this and other clinical settings that POC testing may be useful [74]. It has been estimated that 10–25% of all patients with rheumatologic disorders visiting emergency departments require hospital admission, and up to one-third of hospitalized patients need intensive care [75–78]. These emergencies may present as a rapidly evolving multisystem organ failure, as a mimic of other conditions such as an infectious disease, or with misleading, deceptively benign clinical signs. In a clinic setting, a negative result of a POC ANA test might offer immediate insight and eliminate the need for ordering other expensive autoantibody profiles or referral to a specialist (Fig. 3). Similar approaches could apply to acute-onset vasculopathies such as granulomatosis with polyangiitis or renal-pulmonary syndrome where a specific POC test based on proteinase 3, myeloperoxidase, and/or glomerular basement membrane antibodies might have value as an adjunct to initiating immediate immunotherapy [79]. Having a high level of suspicion, sufficient clinical knowledge, and a method for detection of circulating autoantibody markers are factors that contribute significantly to a timely diagnosis. The use of specific autoantibody testing for the diagnostic process in an acute clinical setting is growing in demand. Availability of a POC for ANA testing has the potential to change practice patterns, not just in rheumatology but also in primary care [80].

New technologies ranging from fairly simple lateral flow technologies to more sophisticated lab-on-a-chip that deliver rapid, low-cost, highly specific, and quantitative results is a goal of point of care testing. Current POC immunoassay technologies come in various configurations and complexities (reviewed in [74, 81]). Several of the newer biosensors have equaled or surpassed traditional central laboratory methods in performance metrics such as sensitivity, specificity, and especially time to result. Devising reliable assays for measuring a specific antibody in human serum is more difficult than measuring most nonantibody analytes in biological fluids, because any one antibody specificity is usually a tiny fraction of total serum immunoglobulin. Nonspecific binding of immunoglobulin may have impeded the development of reliable antibody biosensors. However, recent and evolving advances in the technologies of immunosensors have provided improving accuracy in quantification and low detection limit in testing for some autoantibodies (i.e., anti-dsDNA) commonly used in rheumatology clinical practice [82, 83]. By virtue of its rapid, bed-side, real-time data collection capability, POC testing has the potential to change the practice of medicine, and rheumatology will not be an exception. Commercialization, regulatory certification, and clinician acceptance of POC autoantibody testing in rheumatology is a work in progress.

## Other technologies

A number of other technologies have been developed for the high-throughput testing of sera for autoantibodies. These include a fully automated ANA screening assay [84], a microbead-based ELISA system [85], and nanobarcode technology [86].

## Conclusion

ANA testing has been in clinical use for more than 60 years and was a major advancement in defining SLE as a disease. Measurements of ANAs and related autoantibodies are now commonly used by rheumatologists and nonspecialist providers for diagnosis and classification of SLE and other SARD. However, limitations of the currently available tests have prompted development of other approaches that would offer greater specificity, prognostic value, cost efficiency, and other advantages. These approaches include multiplexed planar and bead arrays as well as rapid detection methods for POC testing, with small sample volumes. Applications including those that would provide assessment of the risk of disease progression are likely to enter clinical practice in the next decade.

## Abbreviations

ALBIA: Addressable laser bead immunoassay
ANA: Antinuclear antibody
BAFF: B cell activating factor
CIA: Chemiluminescence immunoassay
CSF: Cerebrospinal fluid
DFS70: Dense fine speckles 70
ELISA: Enzyme-linked immunosorbent assay
Fab: Antigen-binding antibody fragment
IFN: Interferon
IIF: Indirect immunofluorescence
ILE: Incomplete lupus erythematosus
LEDGF: Lens epithelium-derived growth factor
LIA: Line immunoassay
LN: Lupus nephritis
NET: Neutrophil extracellular trap
NPSLE: Neuropsychiatric systemic lupus erythematosus
POC: Point of care
SARD: Systemic autoimmune rheumatic diseases
SLE: Systemic lupus erythematosus
